# Room-Temperature Ammonia Sensing Using Polyaniline-Coated Laser-Induced Graphene

**DOI:** 10.3390/s24237832

**Published:** 2024-12-07

**Authors:** José Carlos Santos-Ceballos, Foad Salehnia, Frank Güell, Alfonso Romero, Xavier Vilanova, Eduard Llobet

**Affiliations:** 1 MINOS, School of Engineering, Universitat Rovira i Virgili, Avda. Països Catalans 26, 43007 Tarragona, Spain; josecarlos.santos@urv.cat (J.C.S.-C.); frank.guell@ub.edu (F.G.); alfonsojose.romero@urv.cat (A.R.); xavier.vilanova@urv.cat (X.V.); 2 IU-RESCAT, Research Institute in Sustainability, Climatic Change and Energy Transition, Universitat Rovira i Virgili, Joanot Martorell 15, 43480 Vila-seca, Spain; 3 TecnATox—Centre for Environmental, Food and Toxicological Technology, Universitat Rovira i Virgili, Avda. Països Catalans 26, 43007 Tarragona, Spain; 4 ENFOCAT, Facultat de Física, Universitat de Barcelona, C/Martí I Franquès 1, 08028 Barcelona, Spain

**Keywords:** laser-induced graphene, polyaniline, gas sensing, ammonia

## Abstract

The reliable detection of ammonia at room temperature is crucial for not only maintaining environmental safety but also for reducing the risks of hazardous pollutants. In this study, the electrochemical modification of laser-induced graphene (LIG) with polyaniline (PANI) led to the development of a chemo-resistive nanocomposite (PANI@LIG) for detecting ammonia levels at room temperature. The composite is characterized by field emission scanning electron microscopy, Fourier transforms infrared, and Raman and X-ray photoelectron spectroscopy. This work marks the first utilization of PANI@LIG for gas sensing and introduces a simple but effective approach for fabricating low-cost wearable gas sensors with high sensitivity and flexibility.

## 1. Introduction

Graphene is a carbon nanomaterial with sp^2^ bond structures forming a hexagonal honeycomb arrangement in a two-dimensional (2D) layer [1,2,3]. Due to its excellent electrical and thermal conductivity, high specific surface area, charge density, carrier mobility, strength, flexibility, and robustness, graphene is widely used in electronics applications like sensors, biosensors, batteries, supercapacitors and wearable devices [4,5,6,7,8,9,10,11]. The commonly used graphene synthesis processes, including chemical vapor deposition, micromechanical liquid phase exfoliation, electrochemical exfoliation and oxidation–reduction, involve high costs, complex processes with toxic chemicals, long processing times, difficulties for mass production and integration into flexible electronic devices, making graphene production challenging [12,13,14,15,16,17].

In 2014, Tour et al. introduced a viable alternative technology for graphene synthesis, a one-step simple, scalable, and low-cost method for producing three-dimensional (3D) porous graphene films from commercial polymer films prepared by direct laser writing with a CO_2_ infrared laser; this nanomaterial is known as LIG [18,19,20]. LIG is valuable for its attributes, including high surface area, porosity, mechanical flexibility and excellent electrical conductivity [21]. These micro/nanostructures have hydrophilic/hydrophobic surfaces and outstanding electrochemical performance [19,22]. This material’s simple fabrication process and properties enable its application in various fields, such as microfluidic systems, catalysis systems, water purification systems, electronic devices, sensors and biosensors [21,23,24,25,26,27,28].

In recent years, researchers have used LIG in chemo-resistive gas sensors, serving as electrodes [29,30,31,32,33], as a sensitive layer [34,35], or doped with other nanomaterials like metals, metal oxides (MOXs), transition metal dichalcogenides (TMDs) and conductive polymers [36,37,38,39,40,41,42]. These LIG composites provide the capability to detect multiple gas species like nitrogen oxides (NO_x_) [29,30,31,32,35,37], volatile organic compounds (VOCs) [33], carbon dioxide (CO_2_) [34] and ammonia (NH_3_) [42]. In the case of NH_3_, only few studies have been reported where LIG was used for its detection [43].

NH_3_ measurement has broad applications such as air quality monitoring [44], agricultural and livestock practices regulation [45], human breath analysis for medical diagnostics [46], wastewater monitoring [47], and in the chemical industry [48]. Due to the hazards of NH_3_, it is important to develop a real-time monitoring system to identify potential risks and ensure safety [49,50]. These sensors need to meet conditions such as being low-cost, having high precision, showing durability, and consuming low energy. The ability to detect ammonia at room temperature with no need for heating is important for low-power and portable devices. Traditional sensors often work at high temperatures, which limits their use in wearable and flexible applications.

Polyaniline’s (PANI) ability to switch between its emeraldine and leucoemeraldine forms plays a critical role in its gas sensing performance. Upon exposure to ammonia, the protonation/deprotonation process causes a shift between these oxidation states, altering the electrical conductivity of the material. The emeraldine form (conductive state of PANI) undergoes deprotonation while exposed to NH_3_, resulting in the formation of the leucoemeraldine form of PANI, which reduces the conductivity of the polymer [51,52]. The exploration of PANI as a functional material for gas sensors gained traction in the late 1980s, largely due to its unique doping/de-doping capability via protonation and deprotonation reactions [53]. This intrinsic property of PANI, allowing it to modulate its electrical conductivity in response to acidic or basic environments, makes it an ideal candidate for chemo-resistive sensing applications across various chemical analytes. Early breakthroughs in PANI-based ammonia sensors were reported by Hirata et al. (1994) and Kukla et al. (1996), demonstrating impressive gas response levels. These studies highlighted the material repeatability, room-temperature operation, and high environmental stability [53,54].

While there have been previous efforts to use PANI for ammonia detection, they usually involve complex chemical fabrication procedures or non-scalable fabrication methods. Graphene, as an inorganic material with conjugated π electrons, can be combined with PANI to enhance sensitivity and selectivity at room temperature [55,56,57,58,59]. However, such combinations often involve multi-step syntheses or require additional materials for stability. In contrast to the previous study, our approach utilizes a direct electrochemical deposition of PANI onto LIG electrodes on a flexible substrate for the first time [60]. The choice of LIG as a substrate for PANI arises from its unique properties, including high conductivity, a large surface area for gas interactions, and mechanical flexibility. These characteristics complement PANI’s intrinsic sensitivity to NH_3_, creating a composite material that not only improves scalability and stability but also enables applications in flexible and wearable sensing platforms. This method is easy, cost-effective, and scalable compared to the chemical oxidative polymerization often employed in PANI–graphene composites. Moreover, the LIG itself is fabricated using a one-step laser patterning method, eliminating the need for high-cost, multi-step graphene synthesis methods such as chemical vapor deposition. This strategy provides fast and straightforward integration of the sensing material in flexible substrates suitable for wearable applications. The suggested method not only reduces the complexity of the fabrication process but also allows precise control over the thickness and morphology of the PANI layer via electrochemical polymerization. Based on this concept and our previous experience with LIG composites, this research marks the first time that an electrochemical deposition of PANI has been used to fabricate ammonia gas sensors on LIG electrodes, creating new possibilities for industrial-scale production of low-cost and flexible gas sensors [43].

## 2. Materials and Methods

### 2.1. Fabrication of LIG Electrode

An LIG electrode was fabricated using a CO_2_ pulsed infrared laser system (48-2, SYNRARD), with a wavelength of 10.6 μm and a max power of 25 W. The laser was focused on a 50 μm thick commercial polyimide film through a 74 mm focal length lens, and the laser beam was scanned at 200 mm/s, with a pulse frequency of 12 kHz and power of 12%. These are fundamental parameters in controlling the quality and characteristics of the LIG. They influence the LIG’s conductivity, morphology, and suitability for electrochemical polymerization. These parameters were optimized using a digital twin tool that we previously reported [61], as they are key to controlling LIG quality, conductivity, and morphology [62]. The LIG electrode integrates with an 18 mm^2^ sensing area of a 3D porous graphene layer, Ag-ink-coated contact pads, and LIG connection legs into a single structure.

### 2.2. Electrochemical Deposition of PANI on LIG Electrode

**Figure 1:**
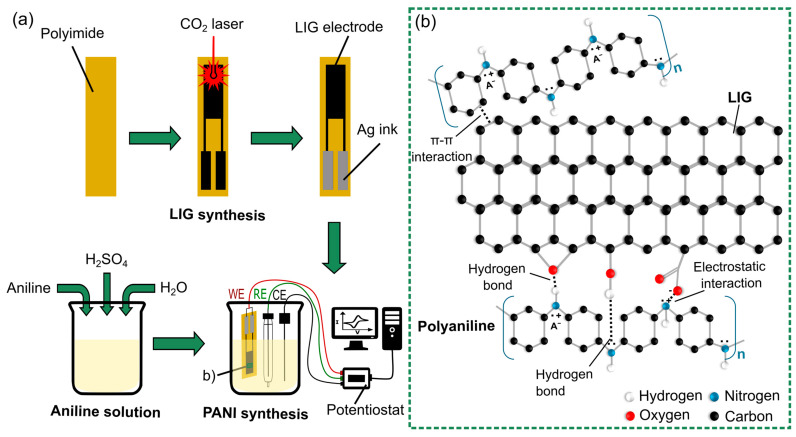
Schematic of (**a**) fabrication process of the PANI@LIG gas sensor and (**b**) interactions between PANI and LIG.

A three-electrode system was used to carry out the electrochemical polymerization process (Figure 1a). The system consists of the LIG electrode as a working electrode, a platinum-wire auxiliary electrode, and an Ag/AgCl reference electrode with a salt bridge containing aqueous 3M NaCl. For the electrochemical polymerization, an aqueous electrolyte containing aniline (Sigma-Aldrich, St. Louis, MO, USA) with a concentration of 0.1 M, along with 1 M H_2_SO_4_ (Sigma-Aldrich), was prepared. The electrochemical polymerization of aniline was performed by using a potentiostat (pocketSTAT2, IVIUM Technologies, Eindhoven, The Netherlands) by cycling the potential between −0.5 and 1.3 V. Figure S1 (Supporting Materials) shows the cyclic voltammograms with a scan rate of 50 mV/s for 20 cycles. The electrodeposited LIG electrode was then rinsed with distilled water and dried in air.

The possible interactions that may occur between aniline–LIG and PANI–LIG are shown in Figure 1b. The XPS analysis shows that the LIG has oxygenated functional groups (such as hydroxyl groups) on the surface and edges of the 3D porous graphene. By these functional groups, aniline monomers can be attached to LIG. Moreover, the LIG has good conductivity, which is required for electrochemical deposition. During the electrochemical deposition, PANI fibers grow on the surface and edges of LIG through electrostatic interaction, hydrogen bonding and π-π stacking between the two components [63]. It is also expected that hydrogen bonds are created between the hydroxyl groups on the LIG and PANI radicals; this causes the attraction of PANI chains on the LIG. The structure of the composite is further stabilized by π-π interaction between the PANI rings and π bonds of LIG [64,65].

### 2.3. Materials Characterization Techniques

Characterizations of the materials were performed in the Scientific and Technical Resources Service (SRCiT) of the University Rovira i Virgil (URV). The Raman analysis of the material was performed using a Renishaw InVia confocal Raman Spectrometer (Wotton-under-Edge, UK) with a coupled confocal microscope (Leica DM2500 Microsystems, Wetzlar, Germany). A 514 nm wavelength laser was employed, with the beam focused onto the LIG surface through a 50× objective lens. A Scios 2 DualBeam field emission scanning electron microscope (FESEM) was used to explore morphology. An optical microscope (Leica DMS300 Microsystems, Wetzlar, Germany). A 514 nm wavelength laser was employed, with the beam focused onto the LIG surface) was employed for visual inspection. The JASCO FT/IR 6700 (Asia portal) spectrophotometer (Tokyo, Japan) was employed for infrared spectroscopy analysis, and X-ray photoelectron spectroscopy (XPS) measurements were performed with ProvenX-NAP, SPECS (Berlin, Germany) using an AlKα—1486.7 eV X-ray monochromatic with µ-FOCUS 600, SPECS source.

### 2.4. Gas Sensing Tests

**Figure 2:**
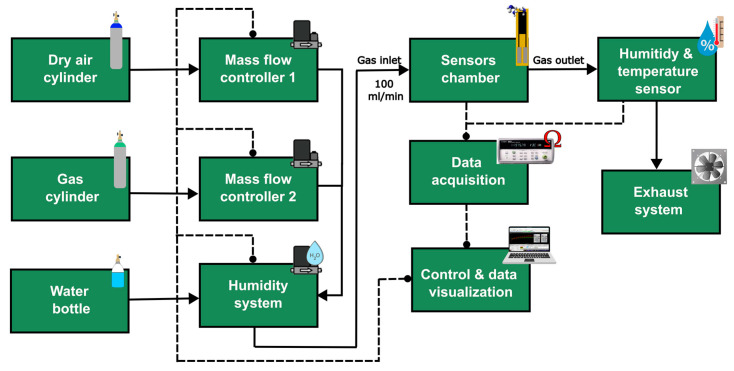
Schematic illustration of measurement system used for gas sensing tests.

The fabricated PANI@LIG sensors were placed in a sealed Teflon chamber (35 cm^3^), to evaluate their gas sensing performance at room temperature (Figure 2). The chamber was isolated from ambient humidity and had the capacity for four sensors. The sensor’s electrical resistance was measured and recorded at a sampling frequency of 0.2 Hz, using a data acquisition system (34972A LXI, Keysight, Santa Rosa, CA, USA) controlled with a PC application (BenchLink Data Logger 3, Agilent Technologies, Santa Clara, CA, USA).

Different gas concentrations were delivered into the chamber with the help of a mass-flow controller (MFC) system (EL-FLOW, Bronkhorst, Ruurlo, The Netherlands), controlled using PC applications (Flow View and Flow Plot, Bronkhorst). This system mixes gases from a zero-grade dry air cylinder as the carrier and a calibrated gas cylinder with 100 ppm of NH_3_ (balanced in dry air) and delivers it to the chamber at a constant rate of 100 mL/min. The sensors were stabilized under dry air for 105 min and then exposed to cyclic exposure of 30 min of NH_3_ (with different concentrations of 5, 10, 25, 50, and 100 ppm) and 75 min of dry air between each concentration. These concentration ranges were selected based on the NH_3_ occupational exposure limits (OELs) according to the European Chemical Agency (ECHA) which specifies a long-term exposure limit (LTEL) of 20 ppm and a short-term exposure limit (STEL) of 50 ppm. The sensor’s relative responses, expressed in percentage, were then calculated as a function of the resistance using the formula Δ*R/R*_0_, where Δ*R* represents *R-R*_0_, *R* corresponds to the value of the resistance after the target gas exposure and *R*_0_ is defined as the sensor’s baseline resistance in air [66,67,68]. The response time (*t_resp_*) and recovery time (*t_recov_*) were calculated as the time to reach 90% of total resistance change for NH_3_ exposure and air re-exposure, respectively [67,68].

The gas sensing performance of sensors under a humid atmosphere was characterized using a controller evaporator mixer (W-202A, Bronkhorst) positioned in series between the gas mixer system and the chamber inlet. In addition, to monitor the environmental conditions during the measurements, a temperature and humidity sensor (SHT85, SENSIRION) was placed at the chamber outlet. The same gas measurement setup was employed to assess the sensor selectivity, replacing the NH_3_ cylinder with a different gas cylinder. Other reducing species were used, including 100 ppm of carbon monoxide, 100 ppm of hydrogen, 20 ppm of ethanol, and aromatic volatile organic compounds such as 10 ppm of benzene and 10 ppm of toluene. Also, the sensor was exposed to 100 ppm of nitrogen dioxide as an oxidizing gas.

## 3. Results and Discussion

### 3.1. Structural and Morphological Characteristics

**Figure 3:**
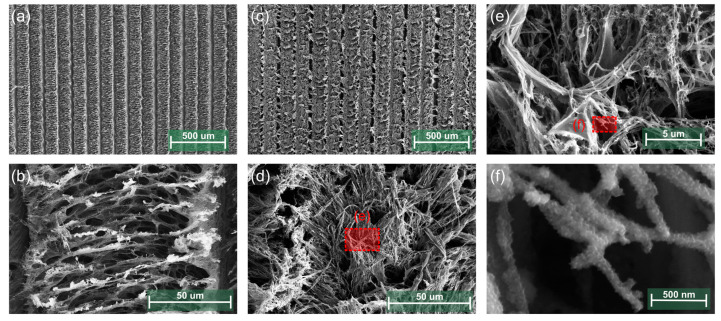
FESEM images of bare LIG (**a**,**b**), PANI@LIG (**c**–**f**).

Figure 3 shows the FESEM images of bare LIG and PANI@LIG. The well-defined porous network seen at both 500 µm and 50 µm scales highlights the rough and interconnected structure of bare LIG demonstrated in Figure 3a,b. It depicts the surface morphology of LIG with a high surface area. The surface of LIG after the electrochemical polymerization is depicted in Figure 3c,d. The surface of the PANI@LIG composite appears to be less dense, with the PANI on the LIG surface covered. This can be seen particularly in the images (d) and (e) where the PANI has been extended in layers upon nanostructures, enhancing the material’s porosity and surface area. Images (e) and (f) further zoom in on the PANI@LIG composite, showing the PANI fibrils forming a fine network at the nanoscale down to 500 nm. Also, Figure S2b,c (Supplementary Materials) comprises optical microscope images of the bare LIG and PANI@LIG, clearly showing the difference between them.

**Figure 4:**
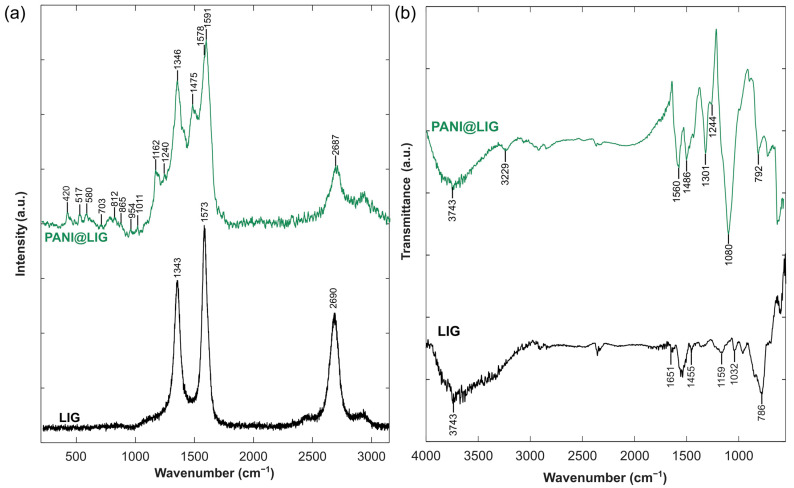
Raman spectra of bare LIG and PANI@LIG (**a**); ATR-FTIR spectra of bare LIG and PANI@LIG (**b**).

The Raman spectra of LIG and PANI@LIG are shown in Figure 4a. Several additional peaks appear in the PANI@LIG spectrum, showing PANI’s successful polymerization on the LIG surface. Key peaks located around 1591, 1475, 1346, and 1162 cm^−1^ were assigned to the C=C stretching of the quinoid and benzenoid rings [69], C–N^+^ stretching modes, confirming the presence of conductive PANI. The peak at 1162 is for C–H bending and stretching of C–N, and some of the peaks in PANI@LIG at the lower frequency range are indicative of different stretching modes and ring deformations which are typical of PANI’s molecular structure. For example, the peaks near 420 cm^−1^ to 580 cm^−1^ are associated with C–N stretching and ring deformation, while peaks near 810 cm^−1^ to 1011 cm^−1^ are linked to C–H out-of-plane bending and in-plane deformations. These low-frequency peaks provide additional evidence for the structural integrity of PANI and confirm its deposition on the LIG substrate. For the LIG spectrum, characteristic peaks are observed near 1573 cm^−1^ and 1343 cm^−1^, which correspond to the G-band and D-band, respectively. The G-band is associated with the in-plane vibrations of sp^2^ carbon atoms in graphitic materials, while the D-band is linked to structural defects and disorder in the graphene structure. Additionally, a peak at approximately 2690 cm^−1^ corresponds to the 2D band, which is another key feature of graphene and its multilayer formation. There is a slight shift in the positions of the G-band and D-band in the PANI@LIG spectrum compared to the pure LIG spectrum. This shift can indicate structural changes or interaction between PANI and LIG through π-π stacking and hydrogen bonding [70].

In order to investigate the infrared absorption properties, FTIR analysis was employed, and the ATR-FTIR spectra are shown in Figure 4b for LIG and PANI@LIG. The LIG sample (the black spectra) exhibits the large band in peak 3743 cm^−1^, which can be attributed to O-H groups [71]. The intense absorption bands around 1651 cm^−1^ are related to C=C stretching vibrations of the aromatic ring [72]. Peaks at 1593 cm^−1^, 1455 cm^−1^ and 1159 cm^−1^ also correspond to different in-plane vibrations or deformation modes [73]. The peaks at around 1032 cm^−1^ correspond to C-O stretching vibrations, indicating characteristics of O-containing functional groups on the LIG surface [60]. The peak at 786 cm^−1^ is associated with C–H out-of-plane bending, which typically occurs in aromatic compounds, and it indicates some residual hydrogen or defects within the graphene structure [74]. The upper green spectra show the PANI@LIG spectrum, indicating distinctive absorption bands which appear to confirm the successful polymerization of PANI. The 3743 cm^−1^ peak associated with the O-H group also appears in this spectrum, as well as the 3229 cm^−1^ peak attributed to the N-H group [75]. Notably, peaks near 1560 cm^−1^, 1486 cm^−1^ and 1244 cm^−1^ correspond to the C=C and C-N stretching of quinoid and benzenoid rings in PANI [76]. Peaks reaching the higher wave number 1080 cm^−1^ with their shoulders are often assigned to N=Q=N stretching [77]. A peak located at 792 cm^−1^ corresponds to the aromatic ring and arises from the out-of-plane bending vibration of C-H [78]. The differences between the spectra of LIG and PANI@LIG, especially the emergence of these specific peaks related to PANI, demonstrate the successful electrochemical polymerization of PANI onto the LIG substrate.

**Figure 5:**
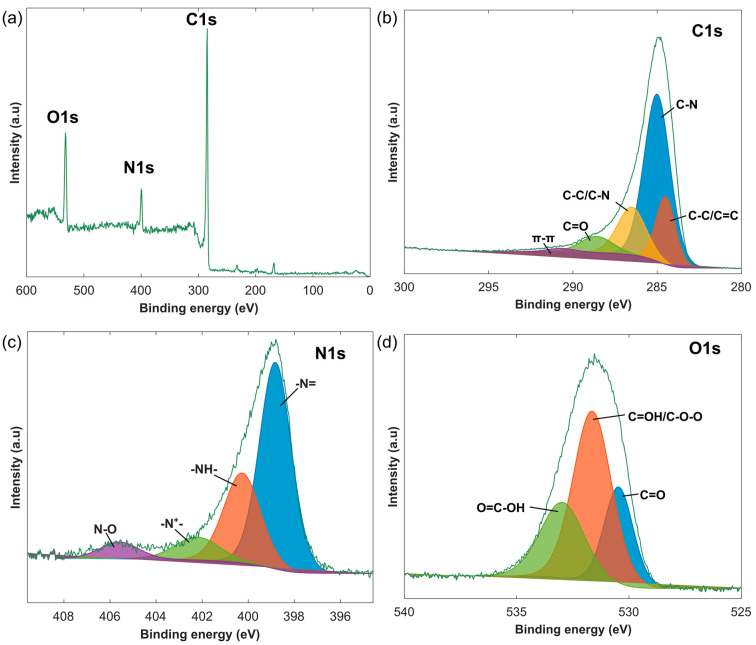
X-ray photoelectron spectroscopy (XPS) survey spectra of PANI@LIG (**a**), and high-resolution spectra fitting results of C1s (**b**), N1s (**c**) and O1s (**d**) of PANI@LIG.

Figure 5a presents XPS analysis, giving us an idea of the surface chemistry of the PANI@LIG composite after being used as sensor. In the high-resolution C1s spectrum, shown in Figure 5b, the C-C/C=C bonds due to sp^2^ hybridized carbon found in the graphene structure are represented by a peak at 284.5 eV [79]. The main peak at 285 eV was found to be associated with C-N bonds and represent PANI on the graphene surface [80]. The peak at 286.5 eV corresponds to C-O or C-N groups and can be attributed to either the oxygenated functional groups on the LIG or carbon–nitrogen interactions in PANI [79]. The peak at 287.3 eV is attributed to carbonyl groups, while the peak at 288.6 eV corresponds to carboxyl groups, more confirmation for the presence of oxygenated species on the LIG [81]. Moreover, the peak at 290.7 eV could be associated with π-π* infarction satellites, suggesting aromatic components in agreement with the conjugation of the graphene structure [79].

The N1s spectrum in Figure 5c further demonstrates nitrogen in the PANI@LIG composite. The peak centered at 398.8 eV is associated with imine groups originated from the structure of PANI, specifically when it adopts its oxidized emeraldine state [81]. The peak at 400.2 eV corresponds to the amine groups, which also originate from the PANI structure [80]. These two peaks confirm the coexistence of the two oxidation states of PANI. The peak at 402.2 eV is assigned to protonated nitrogen species, indicating PANI was in its conductive state known as emeraldine salt [80]. The higher binding energy peak at 405.5 eV is attributed to nitrogen oxide species, which may result from adsorbed NO_2_ while checking the selectivity. The existence of oxygen-containing functional groups on the PANI@LIG composite is further proved by O1s spectrum displayed in Figure 5d. The peak at 530.4 eV is attributed to oxygen in carbonyl groups [82]. The peak at 531.6 eV is assigned to hydroxyl or ether groups, which are typically present in the edges of graphene and give them more water-wettability features [81]. The peak at 532.9 eV is related to carboxyl groups, indicating a further degree of oxidation on the surface [82].

### 3.2. Gas Sensing Performance Analysis

**Figure 6:**
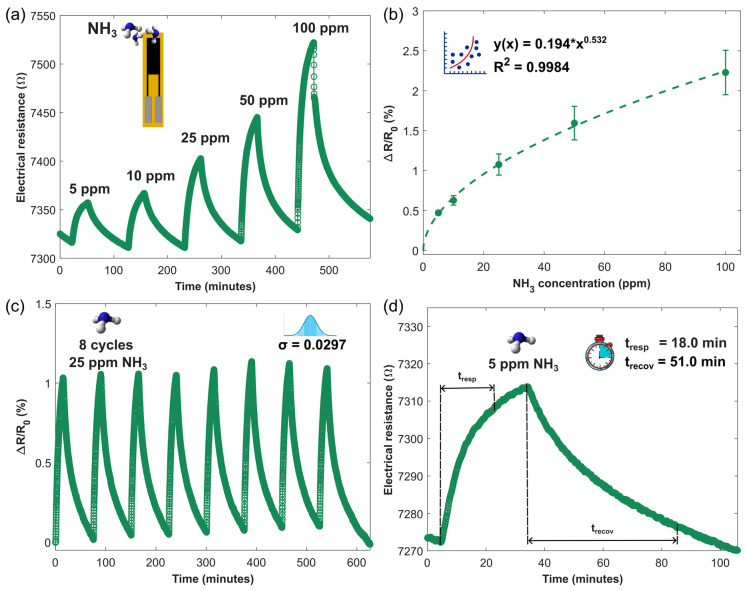
Gas sensing performance of PANI@LIG NCs gas sensors in dry ambient conditions. (**a**) Electrical resistance response to different concentrations (5, 10, 25, 50, and 100 ppm) of NH_3_ at room temperature. (**b**) Regression curve. (**c**) Sensor repeatability testing at successive exposures of 25 ppm of NH_3_. (**d**) Response to 5 ppm of NH_3_ and analysis of response/recovery time.

The gas sensing performance was analyzed at varying concentrations of ammonia with a continuous electrical resistance measurement, in dry ambient conditions. Figure 6a shows the electrical resistance of the PANI@LIG gas sensor increased when exposed to varying concentrations of ammonia. This performance confirms that the PANI@LIG nanocomposite acts as a p-type material on exposure to a reducing gas, with ammonia donating electrons and neutralizing holes (positive charge carriers), thus reducing electrical conductivity. Moreover, the figure highlights the sensor’s baseline stability and minimal noise levels. Figure 6b depicts the sensor regression model as a power function (*y*(*x*) *=* 0.194*x ^0.532^) of the calibration curve. This model was used to calculate the theoretical limit of detection (LOD), following a standard method consistent with the IUPAC definition. According to IUPAC, the LOD is the smallest concentration or absolute amount of analyte that has a signal significantly larger than the signal from a suitable blank. It is calculated based on Equation (1):

(1) $$x_{L}={\bar{x}}_{B}+ks_{B}$$

where ${\bar{x}}_{B}\;$is the mean value of blank measurements (100 baseline points of the sensor’s relative response were analyzed), $s_{B}$ is the standard deviation of the blank measures, and k is a numerical factor (k= 3 for commonly used level of confidence = 99.7%). Then, LOD is estimated by Equation (2) as follows:

(2) $$LOD={\left(ks_{B}/a\right)}^{1/n}$$

where a is the proportionality constant, and *n* is the exponent from the power regression curve. The PANI@LIG sensor shows an LOD of 2.38 ppb for NH_3_. This sensitivity demonstrates the potential efficacy of PANI@LIG for low-concentration detection, comparable to other high-performance materials used in gas sensing applications.

Figure 6c shows the sensor repeatability; it was evaluated by applying eight successive cycles of 25 ppm of NH_3_ for 30 min and recovery steps of 75 min between gas exposures, in which the PANI@LIG sensors present a standard deviation of about 0.029%. Figure 6d indicates the response time (*t_resp_* = 18.0 min) and recovery time (*t_recov_* = 51.0 min) of the sensor to 5 ppm of NH_3_. Considering that this concentration is below the STEL defined by ECHA and no fast detectors are required, a response time in the order of minutes is sufficient. It can be concluded that these times are adequate for this sensor to be used in real-time monitoring of the environment.

**Figure 7:**
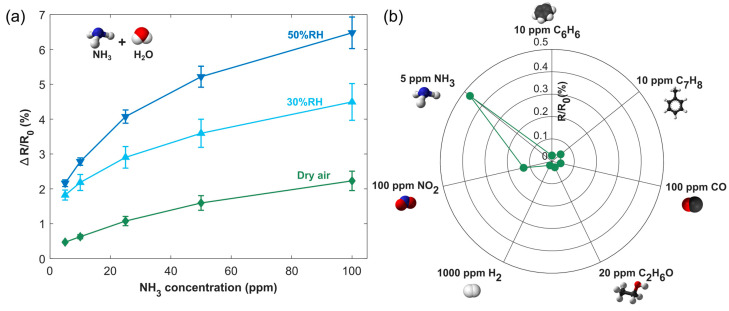
Calibration curves obtained for dry ambient conditions, 30%RH and 50%RH (**a**) and responses to different gas compounds (CO, C_2_H_6_O, C_6_H_6_, C_7_H_8_, NH_3_, H_2_, and NO_2_) (**b**).

Indeed, the influence of environmental moisture on gas sensor performance has emphasized humidity as a key parameter that essentially modulates sensor response. An experiment was carried out under 50% and 30% relative humidity (RH) levels, and the results were compared with those obtained under dry conditions. Figure S3b (Supporting Materials) indicates that variations in RH affect the sensor resistance. As RH increases, the sensor baseline decreases. This effect may be attributed to the further protonation of PANI through absorbed water or the generation of conductive H_3_O^+^. Figure 7a shows that the sensor’s sensitivity improves with an increase in RH. Generally, the sensor response at 50% RH was greater than the responses at 30% RH and in dry conditions. The sensor response for 100 ppm at 50% RH was 2.9 times higher than in dry air. This performance hints that moisture promotes the NH_3_ adsorption on the sensor’s surface. This probably occurs through swelling of the active material by water molecules, causing conductive domains to move further apart, thus leading to higher overall resistance in the film [83]. Additionally, the humid environment may facilitate more efficient proton exchange between the water molecules and NH_3_, further amplifying the sensor’s response. Consequently, an increase in humidity will lead to a higher sensitivity for ammonium detection. Furthermore, sensor response/recovery times (Figure S3b) also improve (decrease) with rising RH.

Selectivity is an important parameter to be considered in gas detection since it demonstrates the ability of the sensor to discriminate the target gas from interfering gases The selectivity was assessed (Figure 7b) by measuring high concentrations of other gases (benzene, toluene, carbon monoxide, ethanol, hydrogen, nitrogen dioxide). Therefore, the PANI@LIG gas sensor exhibited lower responses to these analytes compared with 5 ppm NH_3_, and the fabricated PANI@LIG gas sensors showed acceptable performances for potential detection of environmental ammonia.

The sensor response was checked 45 days after the first test. During this period, the sensors were regularly exposed to ammonia and changing humidity conditions, and were exposed towards different gas species for selectivity tests. Figure S4a (Supporting Materials) shows the long-term stability of responses towards 50 ppm ammonia at room temperature. The sensor response decreased by 30% from day 1 to day 45. Also, the evolution of the baseline resistance can be found in Figure S4b, where the resistance value increases by 3%. We perceive that the decrease in the sensor response is due to the aging of the surface.

Table S1 (Supporting Materials) presents a comparison of various NH_3_ gas sensors reported in the literature, all utilizing graphene–PANI nanocomposites capable of operating at room temperature. In general, the sensing performance was increased when the PANI was present in the sensitive material. However, many studies lack crucial details such as the applied flow rate, the type of carrier gas, and the effect of the influence of ambient humidity on performance. Although our sensor showed a lower response in comparison to those of other studies, its low LOD of the 2.38 ppb combined with the advantage of the electrochemical polymerization and a simple laser drawing method to produce graphene highlight its novelty. This is a simpler method in comparison to those used in most other studies and offers better control over both the thickness and morphology of PANI. Moreover, the sensor and ammonia detection operate at a lower flow rate and use synthetic air instead of nitrogen as the carrier gas, conditions closer to those needed in real-time monitoring for ammonia. Those experimental parameters are likely to influence the sensor response to some extent since higher flow rates or detection in a nitrogen atmosphere can boost the resistance changes.

### 3.3. Gas Sensing Mechanisms

**Figure 8:**
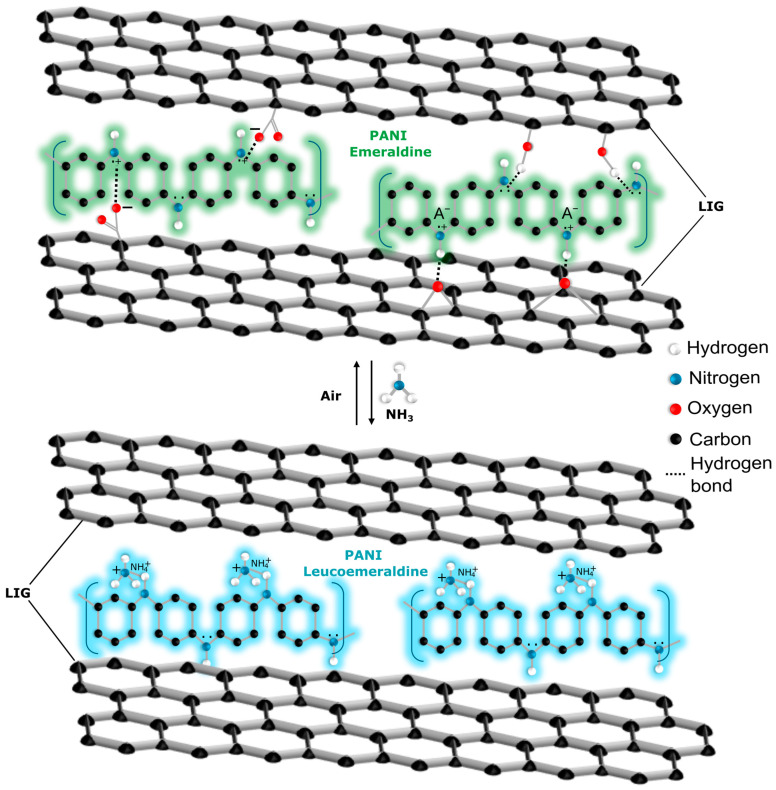
Schematic of the interaction between ammonia and PANI@LIG.

Figure 8 shows the sensing mechanism of ammonia by the PANI@LIG composite. While the sensor is exposed to ammonia, the gas molecules are adsorbed on the surface. The porous structure of the PANI@LIG facilitates fast diffusion of ammonia on the sensor. During electrosynthesis in an acidic electrolyte, PANI molecules become protonated and exhibit p-type semiconductor properties as a result. When an ammonia molecule encounters PANI, it absorbs protons from the polymer so that ammonium ions form. This deprotonation changes the electronic structure of PANI, causing an increase in the sensor’s resistance. The process is reversible; returning the sensor to air allows ammonium ions to convert back to ammonia and a proton, restoring the sensor to its original state [84]. The previous studies showed that the composite of graphene and PANI shows significantly enhanced sensing performance compared to pure PANI [85]. The high surface area of LIG increases the number of adsorption sites that are available for gas molecules and improves the sensor’s sensitivity. LIG’s three-dimensional porous structure with defects and high-energy binding sites helps efficient gas diffusion and adsorption. Additionally, the highly conductive nature of LIG sheets provides a rapid carrier transport network, facilitating swift electron transfer within the sensor. Also, π–π interactions between PANI and LIG enhance electron mobility and create a synergistic effect that improves the ammonia sensing capability of the sensor. The I–V curve in Figure S5 (Supporting Materials) shows the linear relationship between the applied voltage and the flowing current through the sensor, which indicates that under the conditions of exposure, the sensor behaves as a resistive device in which an ohmic contact exists between the gas-sensitive film and the electrodes. The decrease in the slope of the curves when exposed to 50 ppm and 100 ppm NH_3_ compared to synthetic air indicates an increase in the resistance of the sensor. It confirms that when NH_3_ interacts with PANI@LIG, the number of charge carriers decreases due to deprotonation, leading to an increase in resistance.

## 4. Conclusions

In summary, a flexible, low-cost and room-temperature-operating ammonia sensor with high sensitivity and selectivity was developed based on a PANI@LIG composite. The suggested method not only reduces the complexity of the fabrication process but also allows precise control over the thickness and morphology of the PANI layer via electrochemical polymerization. Wearable and portable low-cost devices can directly benefit from this simple fabrication process of a PANI/LIG sensor. While some sensors may offer higher response to ammonia, the convenience, low-cost scalability, low LOD of 2.38 ppb, as well as the sensor’s performance in real-world conditions, make this sensor a candidate for applications such as environmental monitoring and industrial safety.

## Data Availability

Data are contained within the article.

## Supplementary Materials

The following supporting information can be downloaded at: https://www.mdpi.com/article/10.3390/s24237832/s1. Figure S1: Cyclic voltammograms; Figure S2: Picture of the PANI@LIG gas sensor and optical images of the bare LIG electrode and the PANI@LIG gas sensor; Figure S3: Relationship between sensor baseline resistance and relative humidity, and the correlation between sensor response/recovery times and relative humidity; Figure S4: long-term stability analysis; Figure S5: Current-voltage (I–V) characteristics of the gas sensor; Table S1: Comparison with previously reported results.

## References

[1] Novoselov K.S., Geim A.K., Morozov S.V., Jiang D., Zhang Y., Dubonos S.V., Grigorieva I.V., Firsov A.A. (2004). Electric Field Effect in Atomically Thin Carbon Films. Science (1979).

[2] Urade A.R., Lahiri I., Suresh K.S. (2023). Graphene Properties, Synthesis and Applications: A Review. JOM.

[3] Mbayachi V.B., Ndayiragije E., Sammani T., Taj S., Mbuta E.R., Khan A.U. (2021). Graphene Synthesis, Characterization and Its Applications: A Review. Results Chem..

[4] Geim A.K. (2009). Graphene: Status and Prospects. Science (1979).

[5] Singh E., Meyyappan M., Nalwa H.S. (2017). Flexible Graphene-Based Wearable Gas and Chemical Sensors. ACS Appl. Mater. Interfaces.

[6] Xiao Y., Pang Y.X., Yan Y., Qian P., Zhao H., Manickam S., Wu T., Pang C.H. (2023). Synthesis and Functionalization of Graphene Materials for Biomedical Applications: Recent Advances, Challenges, and Perspectives. Adv. Sci..

[7] Weiss N.O., Zhou H., Liao L., Liu Y., Jiang S., Huang Y., Duan X. (2012). Graphene: An Emerging Electronic Material. Adv. Mater..

[8] Han T.H., Kim H., Kwon S.J., Lee T.W. (2017). Graphene-Based Flexible Electronic Devices. Mater. Sci. Eng. R Rep..

[9] Liu J., Bao S., Wang X. (2022). Applications of Graphene-Based Materials in Sensors: A Review. Micromachines.

[10] Syam Sundar L., Amin Mir M., Waqar Ashraf M., Djavanroodi F. (2023). Synthesis and Characterization of Graphene and Its Composites for Lithium-Ion Battery Applications: A Comprehensive Review. Alex. Eng. J..

[11] Lakra R., Kumar R., Sahoo P.K., Thatoi D., Soam A. (2021). A Mini-Review: Graphene Based Composites for Supercapacitor Application. Inorg. Chem. Commun..

[12] Whitener K.E., Sheehan P.E. (2014). Graphene Synthesis. Diam. Relat. Mater..

[13] Sun B., Pang J., Cheng Q., Zhang S., Li Y., Zhang C., Sun D., Ibarlucea B., Li Y., Chen D. (2021). Synthesis of Wafer-Scale Graphene with Chemical Vapor Deposition for Electronic Device Applications. Adv. Mater. Technol..

[14] Kumar N., Salehiyan R., Chauke V., Joseph Botlhoko O., Setshedi K., Scriba M., Masukume M., Sinha Ray S. (2021). Top-down Synthesis of Graphene: A Comprehensive Review. FlatChem Chem. Flat Mater..

[15] Gutiérrez-Cruz A., Ruiz-Hernández A.R., Vega-Clemente J.F., Luna-Gazcón D.G., Campos-Delgado J. (2022). A Review of Top-down and Bottom-up Synthesis Methods for the Production of Graphene, Graphene Oxide and Reduced Graphene Oxide. J. Mater. Sci..

[16] Madurani K.A., Suprapto S., Machrita N.I., Bahar S.L., Illiya W., Kurniawan F. (2020). Progress in Graphene Synthesis and Its Application: History, Challenge and the Future Outlook for Research and Industry. ECS J. Solid State Sci. Technol..

[17] Lin L., Peng H., Liu Z. (2019). Synthesis Challenges for Graphene Industry. Nat. Mater..

[18] Lin J., Peng Z., Liu Y., Ruiz-Zepeda F., Ye R., Samuel E.L.G., Yacaman M.J., Yakobson B.I., Tour J.M. (2014). Laser-Induced Porous Graphene Films from Commercial Polymers. Nat. Commun..

[19] Ye R., James D.K., Tour J.M. (2018). Laser-Induced Graphene. Acc. Chem. Res..

[20] Ye R., James D.K., Tour J.M. (2019). Laser-Induced Graphene: From Discovery to Translation. Adv. Mater..

[21] Vivaldi F.M., Dallinger A., Bonini A., Poma N., Sembranti L., Biagini D., Salvo P., Greco F., Di Francesco F. (2021). Three-Dimensional (3D) Laser-Induced Graphene: Structure, Properties, and Application to Chemical Sensing. ACS Appl. Mater. Interfaces.

[22] Duy L.X., Peng Z., Li Y., Zhang J., Ji Y., Tour J.M. (2018). Laser-Induced Graphene Fibers. Carbon..

[23] Cheng L., Guo W., Cao X., Dou Y., Huang L., Song Y., Su J., Zeng Z., Ye R. (2021). Laser-Induced Graphene for Environmental Applications: Progress and Opportunities. Mater. Chem. Front..

[24] Le T.D., Phan H., Kwon S., Park S., Jung Y., Min J., Chun B.J., Yoon H., Ko S.H., Kim S. (2022). Recent Advances in Laser-Induced Graphene: Mechanism, Fabrication, Properties, and Applications in Flexible Electronics. Adv. Funct. Mater..

[25] Movaghgharnezhad S., Kang P. (2024). Laser-Induced Graphene: Synthesis Advances, Structural Tailoring, Enhanced Properties, and Sensing Applications. J. Mater. Chem. C.

[26] Wan Z., Nguyen N.-T., Gao Y., Li Q. (2020). Laser Induced Graphene for Biosensors. Sustain. Mater. Technol..

[27] Zhu J., Huang X., Song W. (2021). Physical and Chemical Sensors on the Basis of Laser-Induced Graphene: Mechanisms, Applications, and Perspectives. ACS Nano.

[28] Wang H., Zhao Z., Liu P., Guo X. (2022). Laser-Induced Graphene Based Flexible Electronic Devices. Biosensors.

[29] Yan W., Yan W., Chen T., Xu J., Tian Q., Ho D. (2020). Size-Tunable Flowerlike MoS_2_ Nanospheres Combined with Laser-Induced Graphene Electrodes for NO_2_ Sensing. ACS Appl. Nano Mater..

[30] Peng Z., Tao L.-Q., Zou S., Zhu C., Wang G., Sun H., Ren T.-L. (2022). A Multi-Functional NO_2_ Gas Monitor and Self-Alarm Based on Laser-Induced Graphene. Chem. Eng. J..

[31] Tseng S.-F., Chen P.-S., Hsu S.-H., Hsiao W.-T., Peng W.-J. (2023). Investigation of Fiber Laser-Induced Porous Graphene Electrodes in Controlled Atmospheres for ZnO Nanorod-Based NO_2_ Gas Sensors. Appl. Surf. Sci..

[32] Soydan G., Ergenc A.F., Alpas A.T., Solak N. (2024). Development of an NO_2_ Gas Sensor Based on Laser-Induced Graphene Operating at Room Temperature. Sensors.

[33] Li D., Shao Y., Zhang Q., Qu M., Ping J., Fu Y., Xie J. (2021). A Flexible Virtual Sensor Array Based on Laser-Induced Graphene and MXene for Detecting Volatile Organic Compounds in Human Breath. Analyst.

[34] Stanford M.G., Yang K., Chyan Y., Kittrell C., Tour J.M. (2019). Laser-Induced Graphene for Flexible and Embeddable Gas Sensors. ACS Nano.

[35] Yang L., Zheng G., Cao Y., Meng C., Li Y., Ji H., Chen X., Niu G., Yan J., Xue Y. (2022). Moisture-Resistant, Stretchable NO_x_ Gas Sensors Based on Laser-Induced Graphene for Environmental Monitoring and Breath Analysis. Microsyst. Nanoeng..

[36] Yang L., Yi N., Zhu J., Cheng Z., Yin X., Zhang X., Zhu H., Cheng H. (2020). Novel Gas Sensing Platform Based on a Stretchable Laser-Induced Graphene Pattern with Self-Heating Capabilities. J. Mater. Chem. A.

[37] Yi N., Cheng Z., Li H., Yang L., Zhu J., Zheng X., Chen Y., Liu Z., Zhu H., Cheng H. (2020). Stretchable, Ultrasensitive, and Low-Temperature NO_2_ Sensors Based on MoS_2_@rGO Nanocomposites. Mater. Today Phys..

[38] Yang L., Ji H., Meng C., Li Y., Zheng G., Chen X., Niu G., Yan J., Xue Y., Guo S. (2022). Intrinsically Breathable and Flexible NO_2_ Gas Sensors Produced by Laser Direct Writing of Self-Assembled Block Copolymers. ACS Appl. Mater. Interfaces.

[39] Zhao J., Yi N., Ding X., Liu S., Zhu J., Castonguay A.C., Gao Y., Zarzar L.D., Cheng H. (2023). In Situ Laser-Assisted Synthesis and Patterning of Graphene Foam Composites as a Flexible Gas Sensing Platform. Chem. Eng. J..

[40] Zhang Q., Zhang F., Liu X., Yue Z., Chen X., Wan Z. (2023). Doping of Laser-Induced Graphene and Its Applications. Adv. Mater. Technol..

[41] Kwak D., Kim H., Jang S., Kim B.G., Cho D., Chang H., Lee J.O. (2024). Investigation of Laser-Induced Graphene (LIG) on a Flexible Substrate and Its Functionalization by Metal Doping for Gas-Sensing Applications. Int. J. Mol. Sci..

[42] Santos-Ceballos J.C., Salehnia F., Romero A., Vilanova X., Llobet E. (2024). Low Cost, Flexible, Room Temperature Gas Sensor: Polypyrrole-Modified Laser-Induced Graphene for Ammonia Detection. IEEE Sens. J..

[43] Santos-Ceballos J.C., Salehnia F., Romero A., Vilanova X. Electrochemical Deposition of Polyaniline on Laser-Induced Graphene for Room Temperature Ammonia Sensing. Proceedings of the EUROSENSORS XXXVI.

[44] Nair A.A., Yu F. (2020). Quantification of Atmospheric Ammonia Concentrations: A Review of Its Measurement and Modeling. Atmosphere.

[45] Insausti M., Timmis R., Kinnersley R., Rufino M.C. (2020). Advances in Sensing Ammonia from Agricultural Sources. Sci. Total Environ..

[46] Lefferts M.J., Castell M.R. (2022). Ammonia Breath Analysis. Sens. Diagn..

[47] Fang X., Guo X., Shi H., Cai Q. (2010). Determination of Ammonia Nitrogen in Wastewater Using Electronic Nose. Proceedings of the 2010 4th International Conference on Bioinformatics and Biomedical Engineering.

[48] Timmer B., Olthuis W., Berg A. (2005). van den Ammonia Sensors and Their Applications—A Review. Sens. Actuators B Chem..

[49] Yuliarti R., Khambali K., Rusmiati R. (2022). Risk Analysis of Exposure to NH_3_ And H_2_S Gas to Workers in The Small Industrial Environment of Magetan Regency in 2021. Int. J. Adv. Health Sci. Technol..

[50] Reis T., Moura P.C., Gonçalves D., Ribeiro P.A., Vassilenko V., Fino M.H., Raposo M. (2024). Ammonia Detection by Electronic Noses for a Safer Work Environment. Sensors.

[51] Tanguy N.R., Thompson M., Yan N. (2018). A Review on Advances in Application of Polyaniline for Ammonia Detection. Sens. Actuators B Chem..

[52] Wen J., Wang S., Feng J., Ma J., Zhang H., Wu P., Li G., Wu Z., Meng F., Li L. (2024). Recent Progress in Polyaniline-Based Chemiresistive Flexible Gas Sensors: Design, Nanostructures, and Composite Materials. J. Mater. Chem. A.

[53] Hirata M., Sun L. (1994). Characteristics of an Organic Semiconductor Polyaniline Film as a Sensor for NH_3_ Gas. Sens. Actuators A Phys..

[54] Kukla A.L., Shirshov Y.M., Piletsky S.A. (1996). Ammonia Sensors Based on Sensitive Polyaniline Films. Sens. Actuators B Chem..

[55] Farooqi B.A., Ashraf A., Farooq U., Ayub K. (2020). Comparative Study on Sensing Abilities of Polyaniline and Graphene Polyaniline Composite Sensors toward Methylamine and Ammonia. Polym. Adv. Technol..

[56] Wu Z., Chen X., Zhu S., Zhou Z., Yao Y., Quan W., Liu B. (2013). Enhanced Sensitivity of Ammonia Sensor Using Graphene/Polyaniline Nanocomposite. Sens. Actuators B Chem..

[57] Guo Y., Wang T., Chen F., Sun X., Li X., Yu Z., Wan P., Chen X. (2016). Hierarchical Graphene–Polyaniline Nanocomposite Films for High-Performance Flexible Electronic Gas Sensors. Nanoscale.

[58] Gavgani J.N., Hasani A., Nouri M., Mahyari M., Salehi A. (2016). Highly Sensitive and Flexible Ammonia Sensor Based on S and N Co-Doped Graphene Quantum Dots/Polyaniline Hybrid at Room Temperature. Sens. Actuators B Chem..

[59] Chang J., Zhang X., Wang Z., Li C., Hu Q., Gao J., Feng L. (2021). Polyaniline-Reduced Graphene Oxide Nanosheets for Room Temperature NH_3_ Detection. ACS Appl. Nano Mater..

[60] Tohidi S., Parhizkar M., Bidadi H., Mohamad-Rezaei R. (2020). Electrodeposition of Polyaniline/Three-Dimensional Reduced Graphene Oxide Hybrid Films for Detection of Ammonia Gas at Room Temperature. IEEE Sens. J..

[61] Santos-Ceballos J.C., Salehnia F., Romero A., Vilanova X. (2024). Application of Digital Twins for Simulation Based Tailoring of Laser Induced Graphene. Sci. Rep..

[62] Santos-Ceballos J.C., Salehnia F., Romero A., Vilanova X. (2024). Using Laser in the Fabrication of Graphene for Gas Sensing: A Digital Twin Approach. J. Laser Micro/Nanoeng..

[63] Xu J., Wang K., Zu S.-Z., Han B.-H., Wei Z. (2010). Hierarchical Nanocomposites of Polyaniline Nanowire Arrays on Graphene Oxide Sheets with Synergistic Effect for Energy Storage. ACS Nano.

[64] Mitra M., Kulsi C., Chatterjee K., Kargupta K., Ganguly S., Banerjee D., Goswami S. (2015). Reduced Graphene Oxide-Polyaniline Composites—Synthesis, Characterization and Optimization for Thermoelectric Applications. RSC Adv..

[65] Wang H., Hao Q., Yang X., Lu L., Wang X. (2010). Effect of Graphene Oxide on the Properties of Its Composite with Polyaniline. ACS Appl. Mater. Interfaces.

[66] Casanova-Cháfer J., García-Aboal R., Atienzar P., Llobet E. (2019). Gas Sensing Properties of Perovskite Decorated Graphene at Room Temperature. Sensors.

[67] Wang Z., Ni L., Zhang X., Feng L. (2023). A Novel Flexible Substrate-Free NH_3_ Sensing Membrane Based on PANI Covered RGO Functionalized Fiber. Sens. Actuators B Chem..

[68] Wu Q., Shen W., Lv D., Chen W., Song W., Tan R. (2021). An Enhanced Flexible Room Temperature Ammonia Gas Sensor Based on GP-PANI/PVDF Multi-Hierarchical Nanocomposite Film. Sens. Actuators B Chem..

[69] Badi N., Khasim S., Roy A.S. (2016). Micro-Raman Spectroscopy and Effective Conductivity Studies of Graphene Nanoplatelets/Polyaniline Composites. J. Mater. Sci. Mater. Electron..

[70] Ji D., Li B., Raj B.T., Li X., Zhang D., Rezeq M., Cantwell W., Zheng L. (2024). In Situ Surface Polymerization of PANI/SWCNT Bilayer Film: Effective Composite for Improving Seebeck Coefficient and Power Factor. Adv. Mater. Interfaces.

[71] Ajeel K.I., Kareem Q.S. (2019). Synthesis and Characteristics of Polyaniline (PANI) Filled by Graphene (PANI/GR) Nano-Films. J. Phys. Conf. Ser..

[72] Yanilmaz M., Dirican M., Asiri A.M., Zhang X. (2019). Flexible Polyaniline-Carbon Nanofiber Supercapacitor Electrodes. J. Energy Storage.

[73] Yang S., Zhu S., Hong R. (2020). Graphene Oxide/Polyaniline Nanocomposites Used in Anticorrosive Coatings for Environmental Protection. Coatings.

[74] Visan A.I., Popescu-Pelin G., Gherasim O., Grumezescu V., Socol M., Zgura I., Florica C., Popescu R.C., Savu D., Holban A.M. (2019). Laser Processed Antimicrobial Nanocomposite Based on Polyaniline Grafted Lignin Loaded with Gentamicin-Functionalized Magnetite. Polymers.

[75] Butoi B., Groza A., Dinca P., Balan A., Barna V. (2017). Morphological and Structural Analysis of Polyaniline and Poly(o-Anisidine) Layers Generated in a DC Glow Discharge Plasma by Using an Oblique Angle Electrode Deposition Configuration. Polymers.

[76] Feng X.M., Li R.M., Ma Y.W., Chen R.F., Shi N.E., Fan Q.L., Huang W. (2011). One-Step Electrochemical Synthesis of Graphene/Polyaniline Composite Film and Its Applications. Adv. Funct. Mater..

[77] Li Z.L., Yang S.K., Song Y., Xu H.Y., Wang Z.Z., Wang W.K., Dang Z., Zhao Y.Q. (2019). In-Situ Modified Titanium Suboxides with Polyaniline/Graphene as Anode to Enhance Biovoltage Production of Microbial Fuel Cell. Int. J. Hydrogen Energy.

[78] Trchová M., Stejskal J. (2011). Polyaniline: The Infrared Spectroscopy of Conducting Polymer Nanotubes (IUPAC Technical Report). Pure Appl. Chem..

[79] Yang X., Qiu Y., Zhang M., Zhang L., Li H. (2021). Facile Fabrication of Polyaniline/Graphene Composite Fibers as Electrodes for Fiber-Shaped Supercapacitors. Appl. Sci..

[80] Oyetade J.A., Machunda R.L., Hilonga A. (2023). Functional Impacts of Polyaniline in Composite Matrix of Photocatalysts: An Instrumental Overview. RSC Adv..

[81] Liu Y., Ma Y., Guang S., Ke F., Xu H. (2015). Polyaniline-Graphene Composites with a Three-Dimensional Array-Based Nanostructure for High-Performance Supercapacitors. Carbon.

[82] Goswami S., Maiti U.N., Maiti S., Nandy S., Mitra M.K., Chattopadhyay K.K. (2011). Preparation of Graphene–Polyaniline Composites by Simple Chemical Procedure and Its Improved Field Emission Properties. Carbon.

[83] Zhang Y., Zhang J., Jiang Y., Duan Z., Liu B., Zhao Q., Wang S., Yuan Z., Tai H. (2020). Ultrasensitive Flexible NH_3_ Gas Sensor Based on Polyaniline/SrGe_4_O_9_ Nanocomposite with Ppt-Level Detection Ability at Room Temperature. Sens. Actuators B Chem..

[84] Kumar L., Rawal I., Kaur A., Annapoorni S. (2017). Flexible Room Temperature Ammonia Sensor Based on Polyaniline. Sens. Actuators B Chem..

[85] Bai S., Zhao Y., Sun J., Tian Y., Luo R., Li D., Chen A. (2015). Ultrasensitive Room Temperature NH_3_ Sensor Based on a Graphene–Polyaniline Hybrid Loaded on PET Thin Film. Chem. Commun..

