# Supplementary material for: Room-Temperature Ammonia Sensing Using Polyaniline-Coated Laser-Induced Graphene
Source: Sensors (Basel). 2024 Dec 7;24(23):7832. doi: 10.3390/s24237832 (PMC11644887; doi:10.3390/s24237832)
Supplement: Supplementary file 1 [file sensors-24-07832-s001.zip › sensors-3337285-supplementary.pdf]

Supplementary Materials

Room Temperature Ammonia Sensing Using Polyaniline-Coated Laser-Induced Graphene

José Carlos Santos-Ceballos [1, 2, 3], Foad Salehnia [1, 2, 3], Frank Güell [1,4], Alfonso Romero [1, 2, 3], Xavier Vilanova [1, 2, 3], Eduard Llobet [1, 2, 3, *]

[1] Universitat Rovira i Virgili, MINOS, School of Engineering, Avda. Països Catalans 26, 43007 Tarragona, Spain
[2] IU-RESCAT, Research Institute in Sustainability, Climatic Change and Energy Transition, Universitat Rovira i Virgili, Joanot Martorell 15, 43480 Vila-seca, Spain
[3] TecnATox - Centre for Environmental, Food and Toxicological Technology, Universitat Rovira i Virgili, Avda. Països Catalans 26, 43007 Tarragona, Spain.
[4] Universitat de Barcelona, ENFOCAT, Facultat de Física, C/Martí I Franquès 1, 08028 Barcelona, Catalunya, Spain
* Correspondence: F.S.: foad.salehnia@urv.cat , E.L.: eduard.llobet@urv.cat

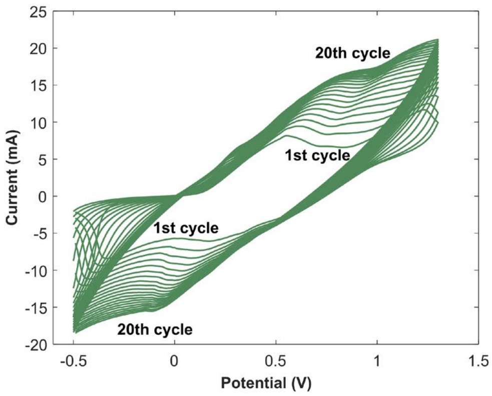

Figure S1. Cyclic voltammograms of the PANI with a scan rate of 50 mV/s for 20 cycles.

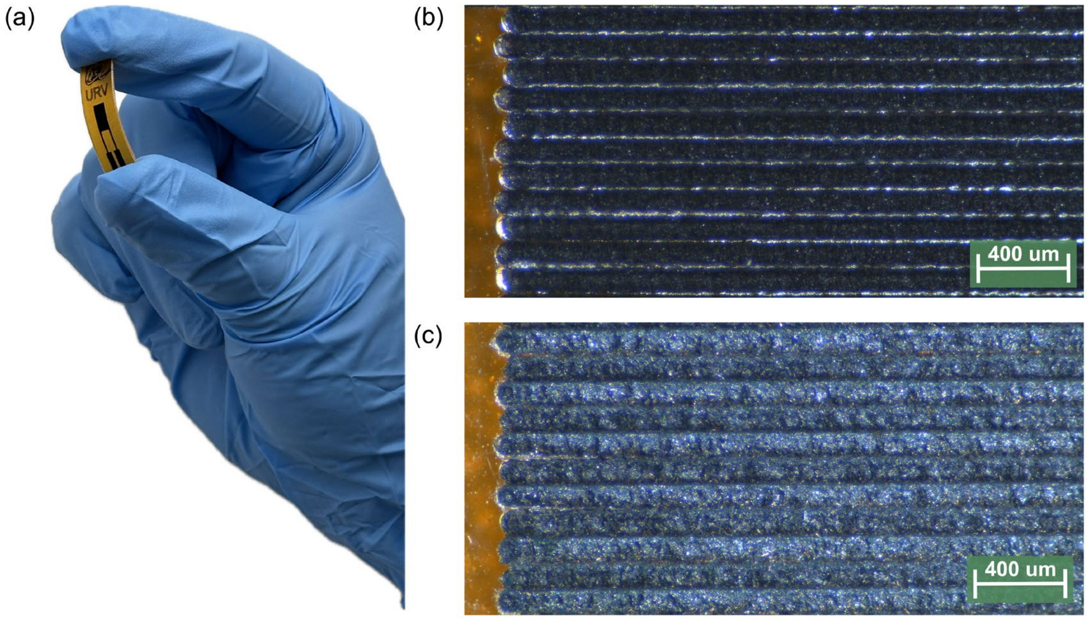

Figure S2. (a) Real picture of the PANI@LIG gas sensor fabricated. Optical images of (b) bare LIG electrode and (c) PANI@LIG gas sensor.

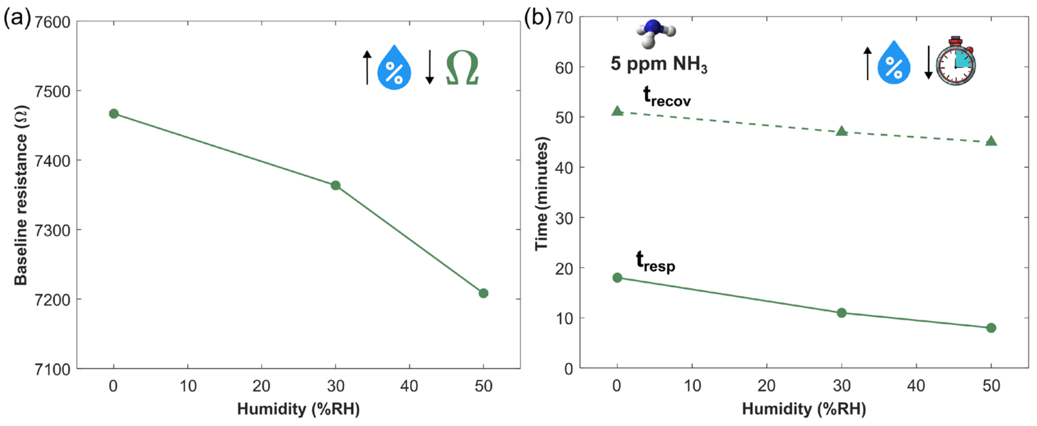

Figure S3. Illustration showing (a) the relationship between sensor baseline resistance and relative humidity, and (b) the correlation between sensor response/recovery times (towards 5 ppm NH_3) and relative humidity.

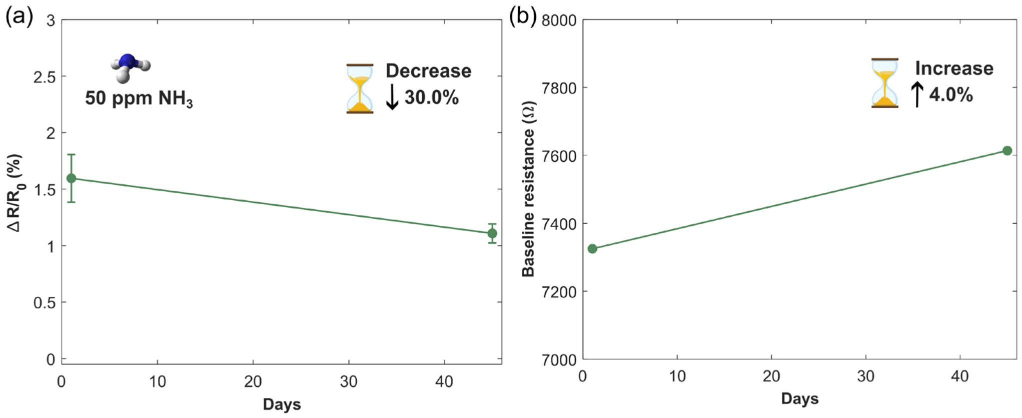

Figure S4. Illustration showing (a) long-term stability study for the sensor responses (5 cycles) towards 50 ppm NH_3 and (b) the evolution of the baseline resistance.

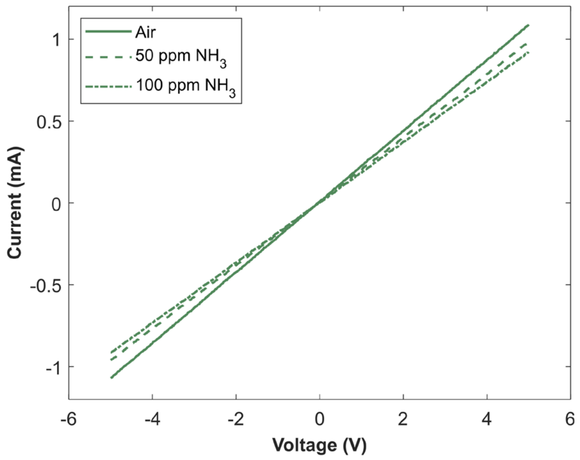

Figure S5. Illustration showing the current-voltage (I-V) characteristics of the gas sensor in air, 50 ppm and 100 ppm NH_3 concentrations.

Table S1 Summary of previous research results for NH_3 sensors operating at room temperature, based on graphene/PANI nanocomposites. G: graphene, PANI: polyaniline, rGO: reduce graphene oxide, GO: graphene oxide

Sensing Material	Polymerization method	Flow (ml/s)	Carrier gas	Humidity (%RH)	Concentration (ppm)	Response (%)	Theoretical LOD (ppm)	Humidity test	Flexible	Reference
3D-rGO/PANI	Electrochemical	-	Air	Dry	5	111	-	No	No	60
N-rGO/PANI	Chemical oxidative	-	-	Ambient	20	200	0.33	Yes	No	86
rGO/PANI	In-situ	-	-	-	100	250	5	No	No	87
rGO/PANI	In-situ	-	-	50	20	30	0.034	Yes	Yes	67
rGO/PANI	In situ	-	-	40	20	1.5	0.046	Yes	No	59
GO/PANI	Ex-situ	-	-	-	5	60	5	No	No	88
GO/PANI	In-situ	-	-	Dry	40	9.6	30	No	No	89
G-PVDF/PANI	In-situ	-	-	70	1	60	0.1	Yes	Yes	68
S, N: GQDs/PANI	/Chemical oxidative	-	Air	57	100	42.3	0.5	Yes	Yes	58
G/PANI	Chemical oxidative	-	N_2	Dry	20	3.65	1	No	No	56
LIG/PANI	Electrochemical	100	Air	50	100	6.46	0.0024	Yes	Yes	This work

References

56. Wu, Z.; Chen, X.; Zhu, S.; Zhou, Z.; Yao, Y.; Quan, W.; Liu, B. Enhanced Sensitivity of Ammonia Sensor Using Graphene/Polyaniline Nanocomposite. *Sens Actuators B Chem* **2013**, *178*, 485–493, doi:10.1016/j.snb.2013.01.014.

58 Gavgani, J.N.; Hasani, A.; Nouri, M.; Mahyari, M.; Salehi, A. Highly Sensitive and Flexible Ammonia Sensor Based on S and N Co-Doped Graphene Quantum Dots/Polyaniline Hybrid at Room Temperature. *Sens Actuators B Chem* **2016**, *229*, 239–248, doi:10.1016/J.SNB.2016.01.086.

59. Chang, J.; Zhang, X.; Wang, Z.; Li, C.; Hu, Q.; Gao, J.; Feng, L. Polyaniline-Reduced Graphene Oxide Nanosheets for Room Temperature NH_3 Detection. *ACS Appl Nano Mater* **2021**, *4*, 5263–5272, doi:10.1021/acsanm.1c00633.

60. Tohidi, S.; Parhizkar, M.; Bidadi, H.; Mohamad-Rezaei, R. Electrodeposition of Polyaniline/Three-Dimensional Reduced Graphene Oxide Hybrid Films for Detection of Ammonia Gas at Room Temperature. *IEEE Sens J* **2020**, *20*, 9660–9667, doi:10.1109/JSEN.2020.2991128.

67. Wang, Z.; Ni, L.; Zhang, X.; Feng, L. A Novel Flexible Substrate-Free NH_3 Sensing Membrane Based on PANI Covered RGO Functionalized Fiber. *Sens Actuators B Chem* **2023**, *380*, 133307, doi:10.1016/j.snb.2023.133307.

68. Wu, Q.; Shen, W.; Lv, D.; Chen, W.; Song, W.; Tan, R. An Enhanced Flexible Room Temperature Ammonia Gas Sensor Based on GP-PANI/PVDF Multi-Hierarchical Nanocomposite Film. *Sens Actuators B Chem* **2021**, *334*, 129630, doi:10.1016/j.snb.2021.129630.

86. Tanguy, N.R.; Arjmand, M.; Yan, N. Nanocomposite of Nitrogen-Doped Graphene/Polyaniline for Enhanced Ammonia Gas Detection. *Adv Mater Interfaces* **2019**, *6*, 1900552, doi:10.1002/admi.201900552.

87. Hadano, F.S.; Gavim, A.E.X.; Stefanelo, J.C.; Gusso, S.L.; Macedo, A.G.; Rodrigues, P.C.; Mohd Yusoff, Abd.R. bin; Schneider, F.K.; Deus, J.F. de; José da Silva, W. NH_3 Sensor Based on RGO-PANI Composite with Improved Sensitivity. *Sensors* **2021**, *21*, 4947, doi:10.3390/s21154947.

88. Borah, S.; Akbar, A.; Das, M.; Sarkar, D. Low Concentration Ammonia Detection at Room Temperature by Polyaniline (PANI)–Graphene Oxide (GO) Composite. *Braz J Phys* **2024**, *54*, 176, doi:10.1007/s13538-024-01524-9.

89. Mohammed, H.Y.; Farea, M.A.; Sayyad, P.W.; Ingle, N.N.; Al-Gahouari, T.; Mahadik, M.M.; Bodkhe, G.A.; Shirsat, S.M.; Shirsat, M.D. Selective and Sensitive Chemiresistive Sensors Based on Polyaniline/Graphene Oxide Nanocomposite: A Cost-Effective Approach. *J Sci: Adv Mater Devices* **2022**, *7*, 100391, doi:10.1016/j.jsamd.2021.08.004.
